# Identification of SPP1 as a Prognostic Biomarker and Immune Cells Modulator in Urothelial Bladder Cancer: A Bioinformatics Analysis

**DOI:** 10.3390/cancers15235704

**Published:** 2023-12-04

**Authors:** Taoufik Nedjadi, Mohamed Eldigire Ahmed, Hifzur R. Ansari, Sihem Aouabdi, Jaudah Al-Maghrabi

**Affiliations:** 1 King Abdullah International Medical Research Centre, King Saud Bin Abdulaziz University for Health Sciences, Ministry of National Guard-Health Affairs, Jeddah 21423, Saudi Arabia; ansarihi@mngha.med.sa (H.R.A.); aouabdisi@mngha.med.sa (S.A.); 2 Faculty of Basic Sciences, King Saud Bin Abdulaziz University for Health Sciences, National Guard-Health Affairs, Jeddah 21423, Saudi Arabia; ahmedmo2@ngha.med.sa; 3 Department of Pathology, Faculty of Medicine, King Abdulaziz University, Jeddah 21589, Saudi Arabia; jalmgrabi@kau.edu.sa; 4 Department of Pathology, King Faisal Specialist Hospital & Research Center, Jeddah 21499, Saudi Arabia

**Keywords:** SPP1, bladder cancer, prognostic, biomarker, bioinformatics, immune infiltration

## Abstract

**Simple Summary:**

Bladder cancer (BC) is one of the most common urological cancers affecting more men than women. The absence of biomarkers for early detection and prognostication add to the burden of the disease. The SPP1 protein is differentially expressed in many malignancies and could serve as a potential prognostic biomarker. However, its expression profile, clinical significance and prognostic value, as well as its relationship with immune infiltration, have not been comprehensively studied in bladder cancer. Herewith, we aimed to investigate the prognostic value of SPP1 expression and its association with other cancer-promoting factors in bladder cancer. Our data indicated that SPP1 is differentially expressed at an early stage of BC development and is associated with an unfavorable prognosis. Interaction between SPP1 and other proteins/genes and immune cell infiltrates may play an important role in the pathogenesis and could as a potential therapeutic target for bladder cancer.

**Abstract:**

Secreted phosphoprotein-1 (SPP1) expression is differentially altered in many malignancies and could serve as a potential prognostic biomarker. Recent findings indicated that SPP1 possesses a broader role in bladder cancer (BC) pathogenesis than previously envisioned; however, the underlying mechanisms governing its expression, cellular localization, prognostic value and immune-related role in bladder cancer remain poorly understood. The expression and the prognosis value of SPP1 were assessed using immunohistochemistry (IHC) staining on a tissue microarray. SPP1 expression was correlated with the clinicopathological parameters, and survival analysis was calculated using a Kaplan–Meier plotter. Bioinformatics analysis of TCGA data was queried using UALCAN, CIBERSORT and TIMER datasets to decipher the biological processes enrichment pattern, protein–protein interactions and characterize tumor-infiltrating immune cells, respectively. IHC revealed that SPP1 expression is significantly associated with tumor type, stage, grade and smoking status. The Kaplan–Meier survival curve showed that low SPP1 expression is an unfavorable prognostic indicator in bladder cancer patients (*p* = 0.02, log-rank). The significant increased expression of the SPP1 level is associated with evident hypomethylation of the gene promoter in cancer compared to normal tissues in the TCGA-bladder dataset. Missense mutation is the most frequent genetic alteration of the *SPP1* gene. Protein–protein interactions demonstrated that SPP1 shares the same network with many important genes and is involved in many signaling pathways and biological processes. TIMER reported a significant correlation between SPP1 expression and multiple immune cells infiltration. Furthermore, the expression of SPP1 was found to be positively correlated with a number of immune checkpoint genes such as PD-1 and CTLA4. The current investigation indicates that the SPP1 protein could serve as a prognostic biomarker and merit further investigation to validate its clinical usefulness in patients with bladder cancer.

## 1. Introduction

Bladder cancer is the second most common urological malignancy after prostate cancer and represents a major cause of high mortality and comorbidity in men [1,2]. Recent reports revealed that the incidence of bladder cancer is increasing at an alarming pace in the developing countries especially in Egypt and Middle Eastern countries [3]. Pathologically, the disease is classified into non-muscle-invasive bladder cancer (NMIBC) and muscle-invasive bladder cancer (MIBC). The overall survival (OS) remains very poor especially for MIBC and metastatic patients with a 5-year OS of less than 50% with radical radiotherapy [4]. Also, the high rates of recurrence and progression to a muscle-invasive phenotype are the main obstacles that compromise the clinical management of the disease [5]. Currently, the assessment of prognosis relies enormously on the conventional staging and grading systems which lack enough accuracy to predict patients’ outcome [6]. This is mainly due to the large molecular heterogeneity of the disease [7]. Altogether, this situation highlights the complexity of the disease and necessitates the urgent need for additional prognostic biomarkers to improve patients’ outcome. The discovery of novel prognostic biomarkers is of paramount importance for high-risk patient stratification in order to identify those who need a more radical therapy at the outset. The molecular stratification would help in setting personalized management approaches and enhance the concept of precision oncology. Several attempts have been made to develop reliable screening biomarkers using urine, tissue or blood-based samples. However, most of them lack sufficient sensitivity and specificity for detecting cancer at an early stage and predicting outcomes [8,9]. Hence, it is crucial to dedicate additional efforts in order to identify novel biomarkers of great clinical significance to improve prognosis, optimize targeted therapy and alleviate the burden of the disease.

Secreted phosphoprotein-1 (SPP1), also known as osteopontin (OPN), is a secreted glycophosphoprotein and a member of the small integrin-binding ligand N-linked glycoproteins (SIBLING) that is encoded by the *SPP1* gene localized on the long arm of chromosome 4q13 [10]. Biologically, secreted SPP1 has been associated with bone regeneration, cell adhesion, migration, invasion, chemotaxis and apoptosis [11]. The cellular functions of SPP1 have also been investigated in cancer and other disorders including obesity, diabetes and cardiovascular disease [12,13,14]. Aberrant over-expression of the SPP1 protein was reported in several solid tumors including prostate cancer [15], lung cancer [16], gastric cancer [17], breast cancer [18] and hepatocellular [19] and it is down-regulated in colorectal and endometrial carcinomas [20,21]. It has been reported that SPP1 regulates the anchorage-independent growth of tumor cells, invasion and chemo-resistance [22]. Interestingly, the expression of SPP1 is highly regulated in invasive cancer cells, indicating its intriguing role in cancer progression and distant metastasis [23]. Furthermore, recent findings reported that SPP1 could mediate the immune response and influence immunotherapy response in lung cancer through the modulation of macrophage M2 polarization and PD-L1 expression [24,25]. In the context of bladder cancer, the involvement of *SPP1* in the pathogenesis of the disease is still poorly understood. Zaravinos et al. (2011) reported differential overexpression of the *SPP1* gene transcript using DNA microarray analysis, while Ke et al. (2011) demonstrated an overexpression of the SPP1 protein in urothelial malignancy using immunohistochemistry [26,27]. Taken together, these findings suggested an important role played by SPP1 cancer development and progression and prompted us to investigate the protein expression patterns and the prognostic value, as well as the molecular pathways and biological function of SPP1. Additionally, we used a combination of online-available datasets and experimental data to comprehensively analyze SPP1-interacting proteins and its potential involvement in immune infiltration in bladder cancer.

## 2. Materials and Methods

### 2.1. Study Population and Sample Collection

A consecutive series of 182 bladder cancer patients, including 152 male patients (83.5%) and 30 female patients (16.5%) with a mean age of 61 years, were investigated. A tissue microarray (TMA) was constructed using formalin fixed paraffin-embedded (FFPE) blocks from consented bladder cancer patients who underwent bladder resection at King Abdulaziz University Hospital, Jeddah (KSA), between 2005 and 2010. None of the patients had received chemotherapy or radiotherapy prior to the surgical resection. The histological variants of bladder cancer include muscle-invasive bladder cancer (MIBC, 43.9%) and non-muscle-invasive bladder cancer (NMIBC, 39%). For correlation analysis, the clinical and pathological data of cancer patients, including age, sex, tumor grade, TNM stage smoking status and other parameters, were collected from patients’ medical records. The histopathological characteristics of the study cohort are described in Table 1a. The median patient age and follow-up was 62 (IQR = 17) and 14.4 months (IQR = 38), respectively (Table 1b).

### 2.2. TCGA Gene Expression Analysis

UALCAN, a web-based tool was used to evaluate the expression of SPP1 across various cancer types, as well as the methylation status of the *SPP1* gene in bladder cancer. These data were extrapolated using UALCAN, a publicly available interactive online portal (http://ualcan.path.uab.edu/index.html accessed on 23 June 2023). The portal enables easy access to the mRNA expression of SPP1 in both cancer tissues and the matching normal counterparts based on The Cancer Genome Atlas (TCGA) database.

### 2.3. Tissue Microarray (TMA) Construction

Cylindrical tissue cores (1 mm in diameter) were extracted from FFPE donor blocks and arranged in duplicate in new recipient blocks. Tissue specimens were sectioned at a thickness of 5 μM, and the slides were then subjected to immunohistochemical staining.

### 2.4. Immunohistochemistry (IHC)

Immunohistochemical staining was performed using the Bench-Mark XT automated system (Ventana Medical Systems, Inc., Tucson, AZ, USA) as previously described [28]. Briefly, after a de-waxing step in xylene and a rehydration step, the FFPE tissue sections were then subjected to antigen retrieval using CC1 conditioning buffer for 60 min. The slides were then incubated with the primary polyclonal human anti-SPP1 antibody (Spring Bioscience, Pleasanton, CA, USA. cat#E3284) at a dilution of 1:100 for 32 min. Subsequently, the slides were treated with the DAB chromogen detection kit, counterstained with hematoxylin then with bluing reagent for 4 min as per the manufacturer’s instructions. After dehydration in graded ethanol and xylene, the sections were permanently mounted with DPX mounting solution and overlaid with coverslips.

### 2.5. Staining Evaluation and Scoring

SPP1 protein expression scoring was performed in a blind fashion to the patients’ clinicopathological data using a 40× objective. The information collected during the scoring process included the sub-cellular localization, the intensity of the staining on scale 0 to 3 (0 = negative; 1 = weak; 2 = moderate; and 3 = strong) and the extent of the staining (percentage of tumor cells showing positive immunoreactivity: 0–100% of cells). A staining index score was calculated by multiplying the staining intensity by the percentage of positive tumor cells [28]. The staining index varied from 0 to 300.

### 2.6. STRING Protein–Protein Interaction (PPI) Analysis

The search for known and predicted protein–protein interaction networks was performed using the STRING database [29]. These data provide evidence on both the functional and physical protein interactions of SPP1. The minimum required interaction score was set at the highest confidence interval (>0.900).

### 2.7. SPP1 Gene Co-Expression Analysis

The molecular alterations of SPP1 in 32 cancer studies were investigated using the TCGA Pan-Cancer Atlas through the cBioPortal cancer genomics website (https://www.cbioportal.org/ accessed on 9 June 2023). We used the expression data from cBioPortal entitled “blca_tcga_pub_2017” which was based on 412 muscle-invasive bladder cancer patients (PMID: 28988769). We used raw RSEM values without normalization. The gene–gene interaction network for humans was downloaded from the BIOGRID (version 4.4.209) database. The expression matrix and interaction network was used in CEMiTool, a Bioconductor package for gene co-expression module identification (PMID: 29458351) with default parameters and filter = TRUE, Pearson correlation and apply_vst (variance stabilizing transformation) = TRUE. We obtained 8 co-expression modules. SPP1 belongs to Module 3 with 468 genes (Supplementary Figure S1). The CEMiTool run gave ~15 most significant hub genes for Module 3. The gene set enrichment analysis was performed by using the clusterProfiler package (PMID: 22455463). All cluster network and KEGG pathway enrichment plots were generated in R.

### 2.8. Analysis of Bladder Cancer Infiltrating Immune Cells

We used the TIMER 2.0 database (PMID: 32442275) for tumor (BLCA)-infiltrating immune cells using SPP1 as the target gene. We selected only significant Spearman’s correlations (*p* < 0.05) with “Purity Adjustment” for bladder cancer.

### 2.9. Relashionship between SPP1 and Immune Checkpoint Genes in BC

The correlation between the SPP1 and immune checkpoint genes was assessed using the cBioPortal database on the TCGA bladder cancer data. Thirty-three immune checkpoint genes were screened and analyzed using the R package.

### 2.10. Statistical Analysis

The correlation between the SPP1 protein expression patterns and patients’ clinicopathological parameters was analyzed using the Chi-square test (χ^2^). For the association between SPP1 levels and overall patients’ survival, univariate survival analysis based on the Kaplan–Meier survival curve was used and the *p*-value was calculated using the log-rank test. The statistical analysis of the data was processed using SPSS (version 21) and data were considered significant for values of *p* < 0.05.

## 3. Results

### 3.1. SPP1 Profiling and Molecular Alterations in TCGA Dataset

**Figure 1:**
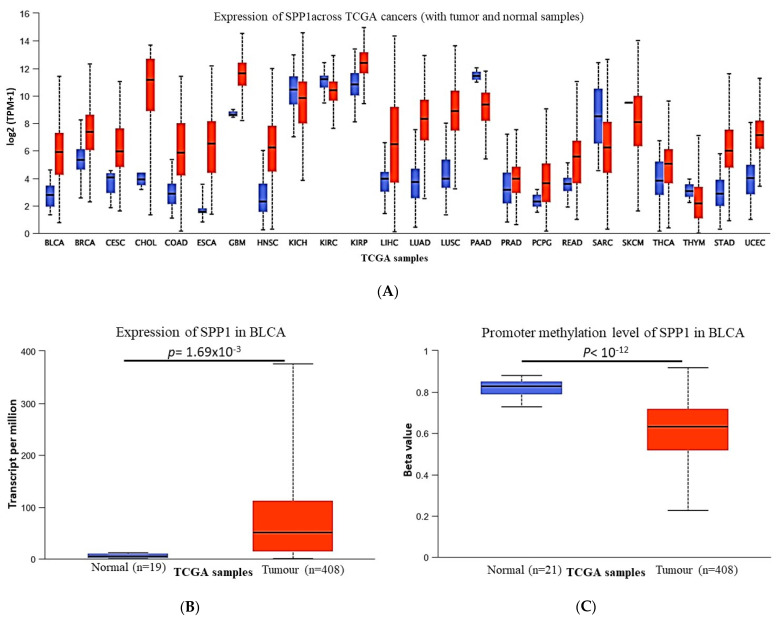
Expression pattern of SPP1 in pan-cancer. (**A**) The expression of the SPP1 mRNA level in multiple TCGA cancers and matching normal tissues. *p* < 0.001, except Thym cancer (*p* = 0.7) and KICH cancer (*p* = 0.53). (**B**) Increased mRNA expression level of SPP1 in bladder cancer. (**C**) Promoter methylation status of SPP1 in bladder cancer and matching normal tissues. All data were analyzed using the UALCAN web tool.

**Figure 2:**
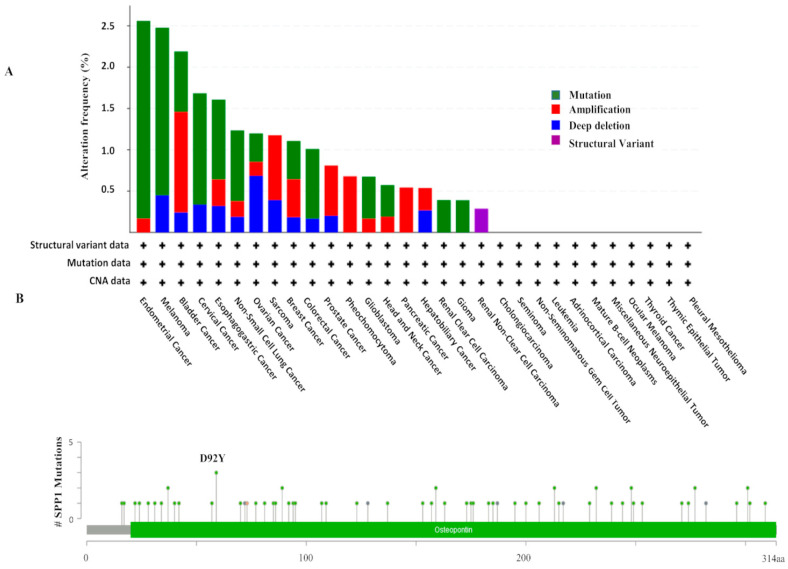
Molecular alterations of SPP1 in cancers. (**A**) High amplifications/mutations of the *SPP1* gene in bladder cancer compared other cancer types. (**B**) Positions and mutation frequency in SPP1 in bladder cancer.

We initially looked at the gene expression profile of SPP1 mRNA levels across a panel of multiple primary cancers and their matching normal tissues (24 in total) using the TCGA database. The data indicated no clear pattern of SPP1 expression, while the SPP1 mRNA level is over-expressed in the common cancers including breast, colon and liver, and we noticed a significant down-regulation of SPP1 expression in renal, pancreatic, cardiac and cartilage cancers compared to the normal tissues (Figure 1A). Compared with normal bladder tissues, bladder cancer showed an increased expression level of SPP1 concomitantly associated with decreased promoter methylation of the *SPP1* gene (Figure 1B,C). The molecular changes in SPP1 were investigated using the cBioPortal for cancer genomics. The genetic alterations range from mutations and amplification to deep deletions. The data indicated that the *SPP1* gene was altered in 2.19% of 411 bladder cases which were found to harbor the highest proportion of *SPP1* gene amplifications with 1.22% relative to other cancer types (Figure 2A). The mutation frequency was also considerable (0.73%), most of which were missense mutations. These included D95Y and R248W, as well as an X73 splice (Figure 2B).

### 3.2. Expression of SPP1 in Bladder Cancer Patients

**Figure 3:**
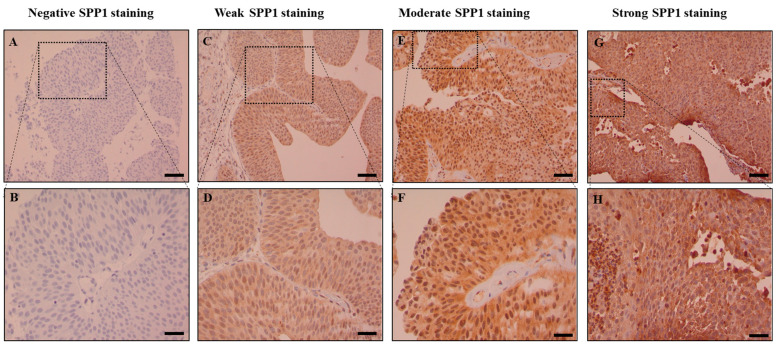
Cytoplasmic expression of SPP1 in bladder carcinoma. Immunohistochemical staining of the bladder cancer tissue microarray using an SPP1 antibody. Figures showing: no expression (**A**,**B**), weak (**C**,**D**), moderate (**E**,**F**) and strong expression (**G**,**H**) of SPP1. Images were taken using 10× and 40× magnification objectives (scale bar equals 1 mm).

**Figure 4:**
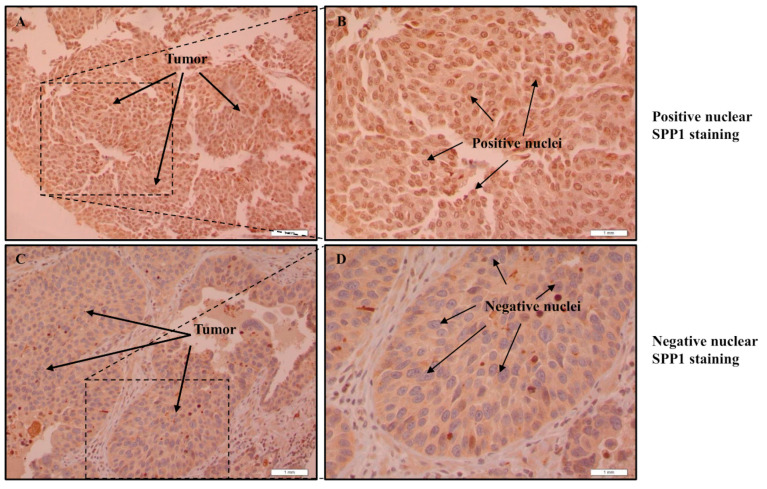
Nuclear SPP1 expression in bladder carcinoma. Immunohistochemical staining of the bladder cancer tissue microarray using an SPP1 antibody. Figures showing: no expression (**C**,**D**), and strong expression of SPP1 (**A**,**B**). Images were taken with 10× and 40× magnification objectives (scale bar equals 1 mm).

The TCGA data indicated that SPP1 may play an important role in bladder cancer pathogenesis and prompted us to investigate the expression pattern and analyze the prognostic value of SPP1 in bladder cancer. In order to do so, tissue microarrays were constructed from 182 Saudi bladder cancer patients and immunohistochemistry staining was performed using an antibody against SPP1. The IHC staining showed that our target protein exhibits both cytoplasmic and nuclear localization. Tumor cells showed diffuse cytoplasmic patterns of staining. The cytoplasmic staining intensity (Table 1c) varied from negative expression (12.8%) to weak expression (60.5%) to moderate expression (25%) to strong expression (1.7%) in some cores (Figure 3). On the other hand, the nuclear SPP1 staining was less prominent compared to the cytoplasmic staining where the expression pattern was observed as follows: 18.9% of the cores were negative, 73.3% exhibited weak staining and 7.8% moderate staining (Figure 4). For correlation analysis, the median intensity was taken as the cut-off point for low and high staining. Therefore, our data indicated that the cytoplasmic positivity of SPP1 staining was detected in 13% of all analyzed cores. For nuclear staining, 92% of the analyzed cohort were scored as low whereas only 8.0% of the specimens were considered as high. Interestingly, a positive association was observed between the staining of SPP1 in the cytoplasmic and nuclear compartments, meaning that patients who expressed high nuclear SPP1 had significantly higher cytoplasmic SPP1 scores (*p* < 0.001) (Supplementary Figure S2).

### 3.3. Relationship between SPP1 Expression and Clinicopathological Characteristics

Next, we sought to analyze the association between SPP1 expression and patients’ clinicopathological features. Our data indicated no association between the cytoplasmic SPP1 score and age, gender and marital status (*p* > 0.05). However, significant correlations were observed between the cytoplasmic level of SPP1 and the tumor grade (*p* = 0.046), stage (*p* = 0.018) and lymph node (*p* = 0.047) (Table 2). Interestingly, a signification positive correlation was apparent between SPP1 expression and smoking status (*p* = 0.002). Furthermore, non-muscle-invasive bladder cancer patients (NMIBC) showed a significant high expression of SPP1 (*p* = 0.019), supporting that increased expression of SPP1 could be an early event in bladder cancer development. Moreover, patients with high nuclear SPP1 expression have a low tumor grade (*p* = 0.007).

### 3.4. Correlation of SPP1 Expression and Survival Outcomes

**Figure 5:**
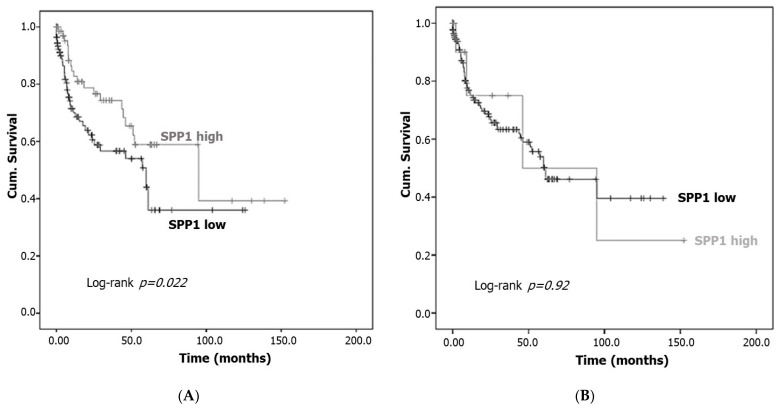
SPP1 expression and patients’ survival. Kaplan–Meier survival curve for bladder cancer patients expressing cytoplasmic (**A**) and nuclear (**B**) patterns of SPP1 (low expression vs. high expression). Low SPP1 immunostaining is associated with poor overall survival (log-rank *p* = 0.022).

The Kaplan–Meier survival curve demonstrated that the reduced expression of cytoplasmic SPP1 is significantly associated with poor patient outcomes (log-rank, *p* = 0.022). The median survival of patients exhibiting high cytoplasmic SPP1 staining was 29 months (95% confidence interval, 16.5–41.5 months), whereas the median survival of patients showing low cytoplasmic SPP1 staining was 35.2 months (95% confidence interval, 24.5–46 months) (Figure 5A). No survival difference was observed for bladder cancer patients when nuclear SPP1 expression was taken in consideration (Figure 5B).

### 3.5. Gene Enrichment and Functional Interactions of SPP1

**Figure 6:**
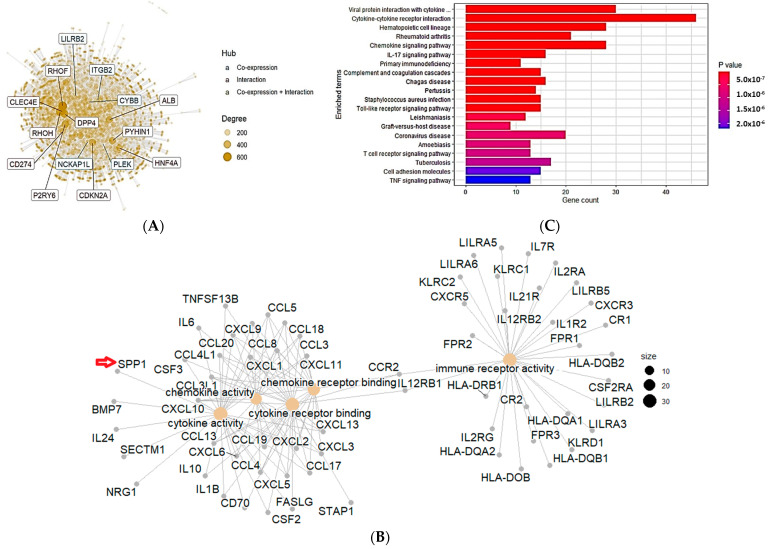
Enrichment analysis of SPP1 in bladder cancer. (**A**) Identification of SPP1-interacting genes. Protein–protein interaction map and hub genes of SPP1. The size of the hub is proportional to the expression level. (**B**) Identification of the SPP1 co-expression network. The figure was generated using the online cBioPortal database. (**C**) KEGG functional enrichment analysis of SPP1. The figure was generated using the online cBioPortal database.

**Figure 7:**
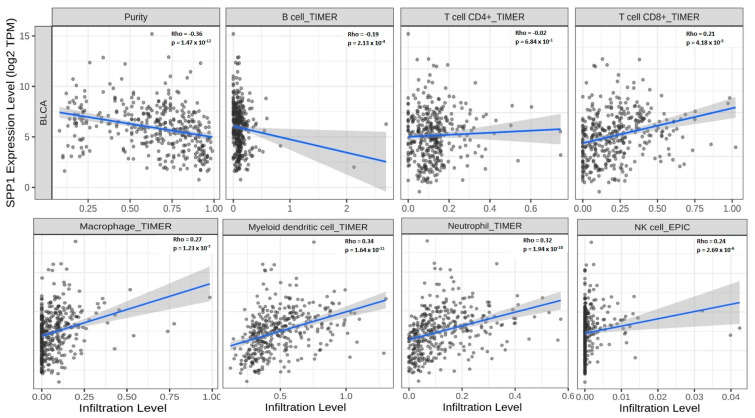
Correlation between immune cells and SPP1 expression. TIMER analysis of the correlation between SPP1 expression and immune cells’ infiltration. Purity-adjusted Spearman’s rho across various cell types by different algorithms.

We next sought to investigate the mechanism by which SPP1 could promote tumor development in bladder cancer. The search for known and predicted protein–protein interactions was performed using the cBioPortal database on TGCA data. A comprehensive quantitative network of SPP1 interactors indicated that our protein of interest belongs to the M3 node which embraces several interacting and co-expressing proteins (Figure 6A). Furthermore, a protein–protein interaction analysis obtained from the STRING website showed a direct interaction between SPP1 and CD44, MMP3, MMP7, TIMP1 and fibronectin-1, all of which are directly involved in the extracellular matrix remodeling and promotion of tumor metastasis [30] (Supplementary Figure S3A). The results revealed that SPP1 could bind to the afore-mentioned network of proteins with the highest confidence value (0.900). Additionally, SPP1 can also recognize albumin (ALB), integrin-A (ITGAV) and integrin-B (ITGB1) proteins whose expression was found to be highly increased at an early stage of bladder cancer development, using the TCGA-BLCA search engine (Supplementary Figure S3B–E). For SPP1-interacting proteins, the analysis of molecular function demonstrated that it is linked to several groups of proteins that are involved in chemokine receptor binding, chemokine activity, cytokine activity and immune receptor activity (Figure 6B). Moreover, KEGG analysis was performed in order to obtain a comprehensive understanding on the molecular pathway mechanisms, as well as the functional annotations associated with SPP1. KEGG gene enrichment analysis demonstrated that SPP1 is mainly enriched in cytokine receptor interaction, chemokine signaling pathway, hematopoietic cell lineage and the IL-17 and TLR signaling pathways (Figure 6C). Using the TIMER, we analyzed the correlation between SPP1 expression and immune cells’ infiltration. As shown in Figure 7, our data indicated that SPP1 expression was positively correlated with the infiltration of CD4^+^ memory-activated T cells, CD8^+^ T cells, neutrophils, dendritic cells and both M1 and M2 macrophages. Additionally, a significant inverse correlation was observed between SPP1 expression and B cells, endothelial cells and monocytes.

### 3.6. Correlation between SPP1 and Immune Checkpoint Genes

**Figure 8:**
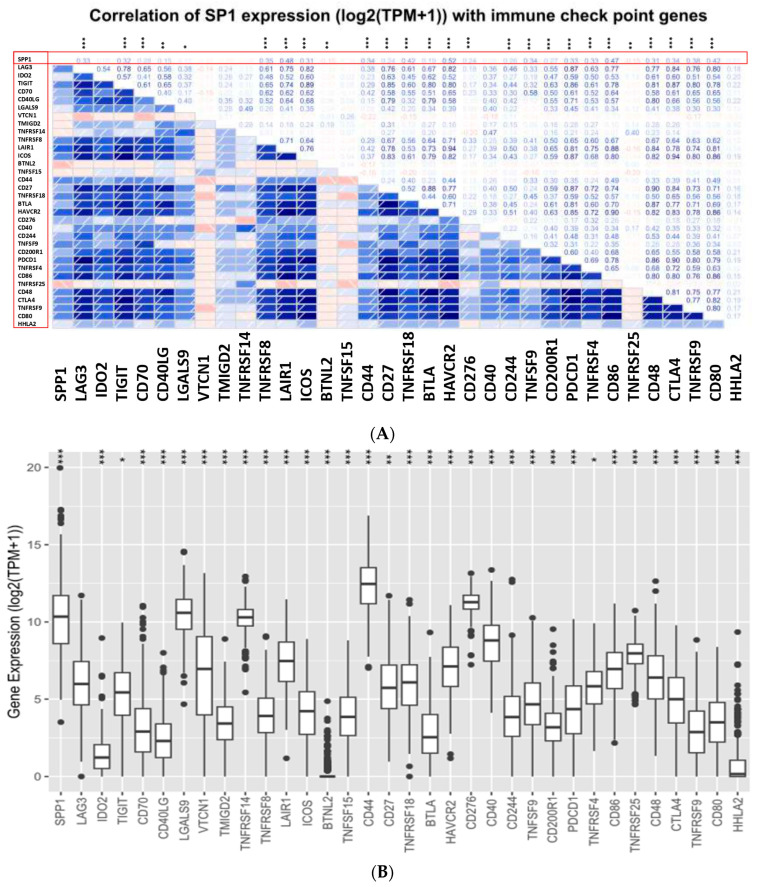
Relationship between SPP1 expression and immune checkpoint genes in bladder cancer. (**A**) Correlation analysis between SPP1 expression and immune checkpoint genes. (**B**) The expression of immune checkpoint genes in relation to SPP1 expression. Data were analyzed using the cBioPortal cancer genomics website on TCGA data. The *p*-value significance codes: *** ≤0.001, ** ≤0.01, * ≤0.05.

Next, we assessed the relationship between SPP1 expression and a list of immune checkpoint genes. Our data indicated that SPP1 expression exhibited a positive association with most of the immune checkpoint genes including PD-1, CTLA4, CD48, BTLA, TIGIT, LAG3 and LAIR1. However, an inverse correlation between SPP1 expression and a few immune checkpoint genes including LGALS9, ICOS, TNFSF1, TNFRSF1 and CD86 was also reported (Figure 8).

## 4. Discussion

Bladder carcinoma is a deadly disease especially when it spreads to the muscle layers of the bladder (MIBC) and the surrounding organs. Recent statistical data indicated that over half a million persons have developed bladder cancer and more than 200,000 patients died of the disease in 2020 [31]. The disease is characterized by its high recurrence rates which necessitate an active long-term surveillance/follow-up regimen [1]. The absence of effective molecular markers for early detection, disease progression and risk stratification renders the therapeutic management of the disease a real challenge. Therefore, new effective early diagnostic and/or prognostic biomarkers that can improve the clinical management of bladder cancer are urgently needed. Thus far, several potential biomarkers have been thoroughly studied [32] and others are currently under investigation, such as SPP1 particularly in bladder cancer. In the present study, we assessed the expression pattern of SPP1 and investigated whether its expression impacts patients’ survival or not. The correlation with the clinical and pathological features in a cohort of Saudi bladder cancer patients was also evaluated. We showed that SPP1 is differentially expressed between healthy controls and disease conditions where the pattern of expression was tissue dependent. The gene expression and DNA methylation of SPP1 from TCGA data demonstrated that the upregulation of SPP1 expression is associated with the decreased methylation of the same gene (Figure 1B,C). In this regard, many studies have linked methylation of the CpG island sites of the promoter region to gene silencing and to alternative gene splicing in cancer [33] which might be the case for the *SPP1* gene in bladder cancer. DNA methylation has been suggested as a biomarker for high-accuracy detection and for outcome prediction in cancer [34]. Interestingly, computational analysis of the TCGA pan-cancer data characterized both gene expression and DNA methylation as the most effective predictors of the somatic mutation state [35], as previously demonstrated with the oncogenic mutations of the IDH1 and IDH2 genes in cancer [36]. The molecular analysis demonstrated that *SPP1* gene amplification was the highest genetic alteration of SPP1 across many cancer types, whereas the frequency of *SPP1* mutation (missense mutation) was low in bladder cancer compared to uterine, skin, lung, cervical, gastric and colorectal cancers. The impact of the *SPP1* gene mutations and/or amplifications on bladder cancer pathogenesis remains to be further verified. It is well documented that bladder cancer is among the tumor types with the highest mutational burden involving several deletions of tumor suppressor genes and the amplification of oncogenes [37]. The amplifications of chromosome 6p22 and 11q are the most common alterations in bladder cancer [38]. Similarly, abnormal amplifications of the *ERBB2*, *mdm2* and *FGFR3* genes were found at a high frequency in bladder cancer [39,40,41]. Alterations of these genes appear to contribute, either directly or indirectly, to the development of bladder cancer and also to have clinical implications in the management of cancer patients.

Our IHC data revealed that high SPP1 expression was observed in around 13.0% of the study cohort, whereas the majority of the study population (87.0%) showed low or no SPP1 expression. Furthermore, patients with lower cytoplasmic SPP1 expression had a significantly reduced survival time compared with those who have higher cytoplasmic SPP1 (Figure 5A). In concordance with our data, reduced expression of SPP1 was reported as an unfavorable prognosticator in both colorectal cancer and endometrial carcinoma [20,21]. These data are also consistent with previous observations by Collins et al. (2012) who demonstrated that higher SPP1 expression is beneficial since it was associated with an improved outcome in patients with pancreatic cancer [42]. By contrast, several other investigations highlighted that high SPP1 levels were correlated with poor outcomes in many solid malignancies such as prostate cancer, lung cancer, gastric cancer and breast cancer [43,44,45,46]. For instance, Wong et al. (2017) showed that the overexpression of SPP1 in bladder cancer patients with high T-stage and tumor grades was significantly associated with decreased patient survival time [47]. The differential expression of SPP1 in the tissue and plasma samples [48] and its association with poor prognosis in cancer patients with an advanced disease stage indicated that SPP1 may serve as a valuable prognostic biomarker in different solid tumors [49,50] and a potential target for cancer therapy [27,51].

Interestingly, the prognostic value of nuclear SPP1 expression in the analyzed cohort could not be established (Figure 5B). However, low cytoplasmic expression of SPP1 correlated with muscle-invasive bladder cancer patients and high cytoplasmic SPP1 expression was predominantly found in patients with non-muscle-invasive bladder cancer (NMIBC) which represents an early stage in bladder cancer development (Table 2). Our results suggested for the first time that high levels of SPP1 may play a role at an early stage of bladder cancer development (NMIBC). Moreover, higher cytoplasmic SPP1 expression was significantly correlated with low-grade tumors and the early tumor stage (Ta) (Table 2). Our data demonstrated that among patients expressing high cytoplasmic SPP1 levels, 78% of them are Stage Ta and T1 and only 22% of the patients have a high tumor stage (T2 and T3). Interestingly, the current data reported the association between the smoking status and the expression level of the cytoplasmic SPP1 protein (*p* = 0.002). These data are in line with a recent study by Jiang et al. (2022) who showed that cigarette smoking increased the expression of OPN through the Jak2/Stat3 pathway in lung cancer [52]. Epidemiological studies demonstrated the strong link between tobacco cigarette smoking and the risk of bladder cancer development [53]. In a meta-analysis conducted on 15 cohort studies, Hou et al. (2017) indicated the strong association between smoking status and the prognosis of patients with bladder cancer [54].

These findings indicate that increased SPP1 expression might be an early event in bladder cancer development. A study by Hussain et al. (2017) reported the up-regulation of cytoplasmic SPP1 expression at a late stage of bladder cancer development [55]. Published reports indicated that cytoplasmic SPP1 is associated with a poor outcome and resistance to radiation via activation of the Jak2-Stat3 pathway. Biologically, SPP1 was reported to play a cancer-promoting role by regulating cell proliferation, motility, invasion and angiogenesis [56,57]. These data indicated that SPP1 could have a significant clinical impact, and patients with higher cytoplasmic levels should be managed differently and monitored carefully. Strikingly, nuclear SPP1 is poorly investigated and not much data are available on the functional role and the clinical implication of the altered expression of nuclear SPP1. The available data indicated that the nuclear isoform of SPP1 (OPN-c) can bind to polo-like kinase-1 and regulate apoptosis through binding to p53 [58]. Furthermore, Zduniak et al. (2015) reported that OPN-c is predominantly expressed in the nucleus in breast cancer and correlated with the tumor grade and a poor outcome in early breast cancer [59]. In our bladder cancer cohort, high nuclear SPP1 expression was seen in only 8.0% of the patients and was significantly associated with a low tumor grade (*p* = 0.007). In colorectal cancer, Assidi et al. (2019) reported a weak nuclear SPP1 expression in 23% of the analyzed cohort. Zduniak et al. (2015) used IHC staining to analyze the expression of SPP1-c which is a splice variant of the *SPP1* gene, in a cohort of 671 breast cancer patients. The investigators revealed a strong staining intensity of nuclear SPP1-c which was significantly associated with poor outcomes in patients with early breast cancer [58]. Altogether, these data suggested that the intracellular localization (cytoplasmic and nuclear) of SPP1 or one of its splice variants merit further investigation, as it might have a clinical impact for cancer patients.

Thus far, the molecular mechanisms that underpin the link between SPP1 expression and bladder cancer progression and outcome remain largely unknown. However, previous studies indicated that SPP1 regulates cancer cells’ proliferation and motility. Using SPP1-targeted siRNA to knock down the levels of SPP1 expression in the T24 cell line, Xu et al. (2015) revealed that SPP1 plays an important role in promoting bladder cancer growth and invasiveness [60]. SPP1 has also been involved in cell adhesion, survival, angiogenesis and metastasis [61]. Recent findings established the link between SPP1 and the epithelial-to-mesenchymal transition (EMT) through the NRP2 protein pathway in bladder cancer cell lines [62]. The involvement of SPP1 in these biological and molecular functions are mainly mediated through binding of multiple cell surface receptors and the activation of various signal transduction pathways that have an impact on all hallmarks of carcinogenesis [63]. For instance, activation of the mitogen-activated protein kinase (MAPK) pathway, mTOR pathway and phosphatidylinositol 3-kinase/Akt signaling pathway have been implicated in OPN-associated cancer development [63]. Moreover, increased evidence supporting the connection between SSP1 and immune cells’ infiltration in promoting cancer progression has been established [64]. Immune checkpoint molecules such as PD-1, PD-L1 and CTLA4, which are targets for cancer immunotherapy, play an important role in maintaining self-tolerance and modulating the cancer immune response [65]. In this study, SPP1 demonstrated a positive correlation with several immune checkpoint genes including PD-1 and CTLA4, suggesting the engagement of SPP1 in carcinogenesis through the recruitment of multiple immune cells and activation of immune-related genes. This process is mediated via binding with integrins and CD44 receptors and activation of the Jak1/Stat1 signaling pathway [66,67]. Recent findings by Zheng et al. (2021) revealed that SPP1 expression was associated with EGFR mutation which conferred resistance to immunotherapy and promoted cancer progression. This pathway was characterized by low CD8^+^ T cells and high M2-type macrophages creating an immunosuppressed microenvironment [24]. In liver cancer, SPP1-positive macrophages had a specific interaction with the fibroblast-associated TME. This distinct communication between these cells promotes the immunosuppressive atmosphere in the TME and facilitates metastatic tumor progression [68]. SPP1-expressing macrophages have been demonstrated to influence the invasive potential of colorectal cancer cells via HLA-G [69]. Emerging data from Bill et al. (2023) found that the expression ratio of SPP1 and CXCL9 is critically important in defining the polarity of macrophages. This ratio has a strong prognostic value as it coordinates a network of pro-tumor variables and determines the antitumor immunity in the TME [70].

Taken together, the current study complements the previous reports on the major role of SPP1 in cancer pathogenesis. SPP1 might be clinically beneficial since its expression is significantly associated with the tumor grade, stage and survival of bladder cancer and other malignancies. Deepti et al. (2022) reported that the SPP1 mRNA level was associated with cancer staging and was a good discriminator between malignant and non-malignant tissues in cervical cancer [71]. This result was consolidated by recent findings indicating that increased SPP1 expression was associated with a poor outcome for patients with lung and breast cancers and promoted drug resistance and EMT transition in prostate cancer [72,73]. Our study sheds light on the prognostic value of SPP1 expression in bladder cancer. Additionally, we also revealed the potential interacting proteins and enrichment pathways that may orchestrate the tumor-promoting role of SPP1. We finally highlighted the association between SPP1 and immune infiltrating cells and immune checkpoint genes in bladder cancer. Given the limitations associated with our study, related mainly to the small sample size, incomplete clinical and pathological patients’ data and the heterogeneity of the disease, the present study highlights the importance of SPP1 as a valuable prognosticator and potential molecular target for bladder cancer therapy.

## 5. Conclusions

The current study sheds light on the importance and the functional significance of SPP1 in bladder cancer. Systematic analysis of data emerging from our cohort and the TCGA dataset revealed that SPP1 may play an important role in cancer development through interaction with several proteins, pathways and TME-associated cells. This work also highlights the importance of allocating additional priority to characterize SPP1 isoforms in cancer concomitantly with clinical research to validate the prognostic value of SPP1 and its potential therapeutic target in bladder cancer in a larger patient cohort and multi-institutional networks.

## Figures and Tables

**Table 1:** (**a**): Baseline clinical and pathological characteristics of bladder cancer patients. (**b**): Median and IQR of patients’ age and follow-up duration. (**c**): Expression intensity of SPP1.

(**a**)				
Variable	n = 182		%	
Gender				
Male	152		83.5	
Female	30		16.5	
Age				
<60	76		41.8	
60+	106		58.2	
Marital status				
Married	174		95.6	
Single	8		4.4	
Type of disease				
MIBC	80		43.9	
NMIBC	71		39.0	
Unknown	31		17.1	
Grade				
Low grade	92		51.1	
High grade	88		48.9	
Stage				
Ta	41		22.5	
1	58		31.8	
2	46		25.3	
3	12		6.3	
4	25		13.7	
Lymph node				
None	158		86.8	
Positive	24		13.2	
Smoking				
Yes	48		26.4	
No	134		73.6	
Status				
Alive	124		68.5	
Dead	57		31.5	
(**b**)				
Variable	Median		IQR	
Age (Years)	62.0		17.0	
Follow-up duration (Months)	14.4		38.0	
(**c**)				
	Staining intensity (%)			
	Negative	Weak	Moderate	Strong
Cytoplasmic	12.8	60.5	25	1.7
Nuclear	18.9	73.3	7.8	0

**Table 2:** Correlation between cytoplasmic SPP1 expression and patients’ clinicopathological characteristics.

Variable	Low 159 (87.4)	High 23 (12.6)	*p* Value
Gender			
Male	130 (81.7)	22 (95.6)	0.093
Female	29 (18.3)	1 (4.4)	
Age			
<60	64 (40.3)	12 (52.2)	0.278
60+	95 (59.7)	11 (47.8)	
Marital status			
Married	151 (94.9)	23 (100)	0.271
Single	8 (5.1)	0 (0)	
Type of disease			
MIBC	75 (47.2)	5 (21.7)	0.019 *
NMIBC	56 (35.2)	15 (65.2)	
Undecided	28 (17.6)	3 (13.1)	
Grade			
Low grade	77 (48.4)	16 (69.9)	0.046 *
High grade	82 (51.6)	7 (30.4)	
Stage			
0	35 (22.0)	6 (26.1)	0.018 *
1	46 (28.9)	12 (52.2)	
2	43 (27.1)	3 (13.0)	
3	10 (6.3)	2 (8.7)	
4	25 (15.7)	0 (0)	
Lymph node			
None	135 (84.9)	23 (100)	0.047 *
Positive	24 (15.1)	0 (0)	
Smoking			
Yes	34 (21.4)	14 (60.9)	0.002 **
No	125 (78.6)	9 (39.1)	
Status			
Alive	106 (67.1)	18 (78.3)	0.281
Dead	52 (32.9)	5 (21.7)	

* *p* < 0.05; ** *p* < 0.01.

## Data Availability

The datasets used and/or analyzed during the current study are available from the corresponding author on reasonable request.

## Supplementary Materials

The following supporting information can be downloaded at: https://www.mdpi.com/article/10.3390/cancers15235704/s1, Figure S1: Module set enrichment. Figure S2: Box plot analysis. Figure S3: Functional interaction of SPP1 in bladder cancer.
