# Supplementary material for: Identification of SPP1 as a Prognostic Biomarker and Immune Cells Modulator in Urothelial Bladder Cancer: A Bioinformatics Analysis
Source: Cancers (Basel). 2023 Dec 4;15(23):5704. doi: 10.3390/cancers15235704 (PMC10705199; doi:10.3390/cancers15235704)
Supplement: Supplementary file 1 [file cancers-15-05704-s001.zip › cancers-2680147-supplementary.pdf]

Supplementary figure 1

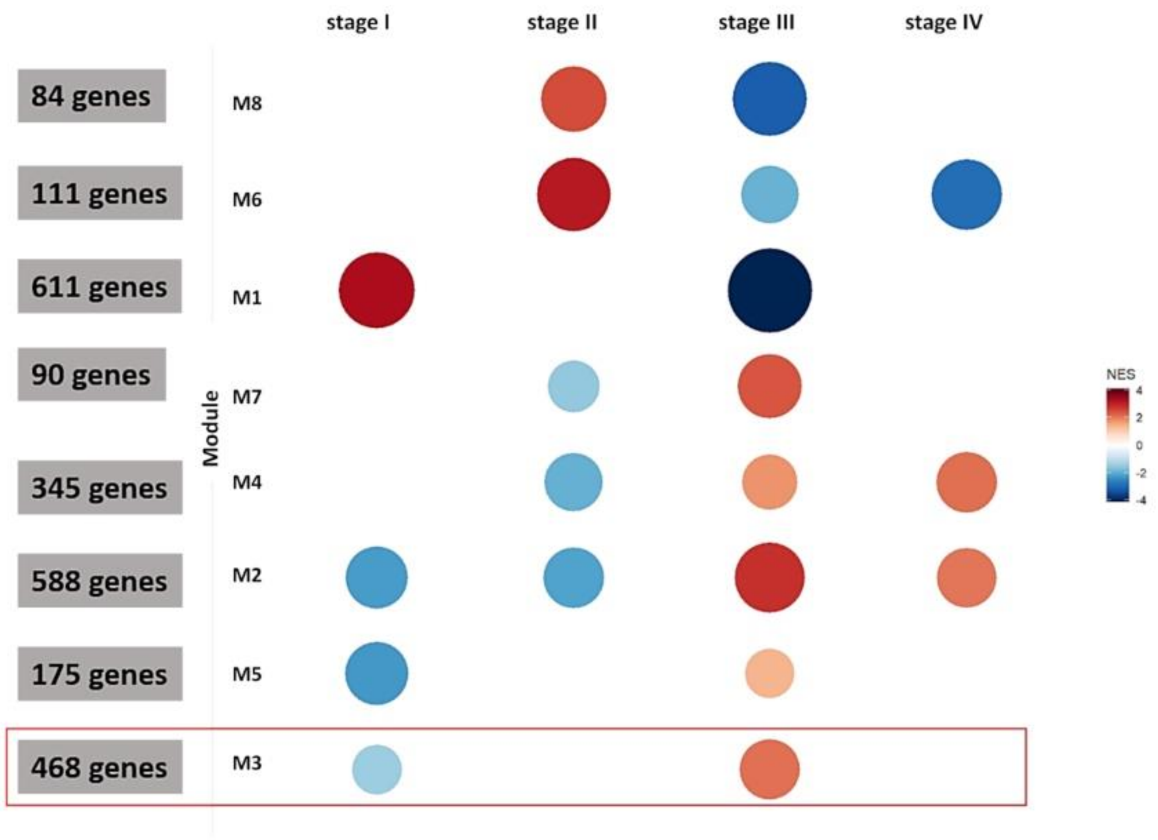

Supplementary figure S1: Module set enrichment. Using a bioconductor package for gene co-expression module identification, CEMiTool, we obtained 8 co-expression modules. SPP1 belongs to module 3 containing 468 genes.

Supplementary Figure 2

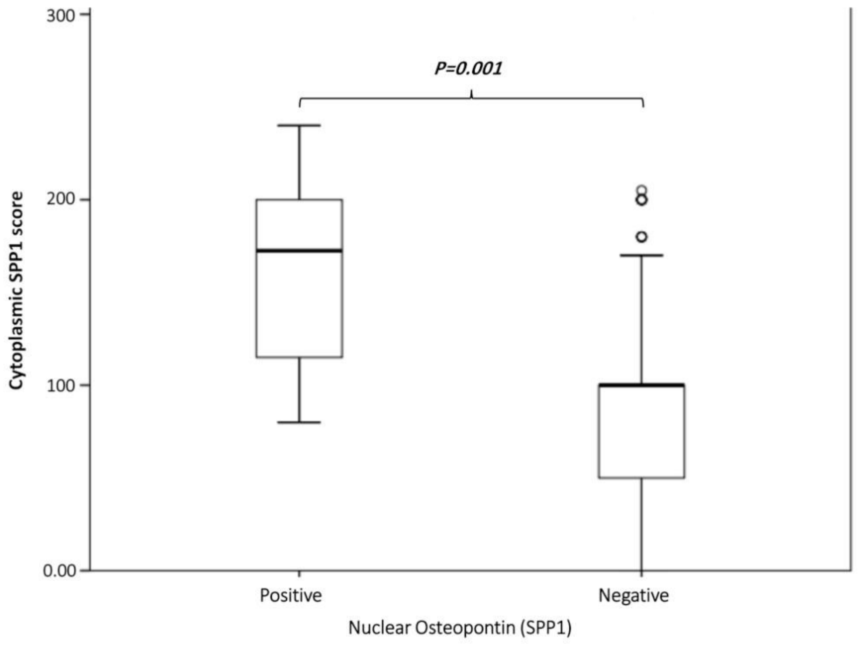

Supplementary figure S2: Box plot analysis. The staining scores for cytoplasmic SPP1 were plotted for the tumors that exhibit positive or negative nuclear SPP1 staining. Significant difference of the median scores was found (*p = 0.001*).

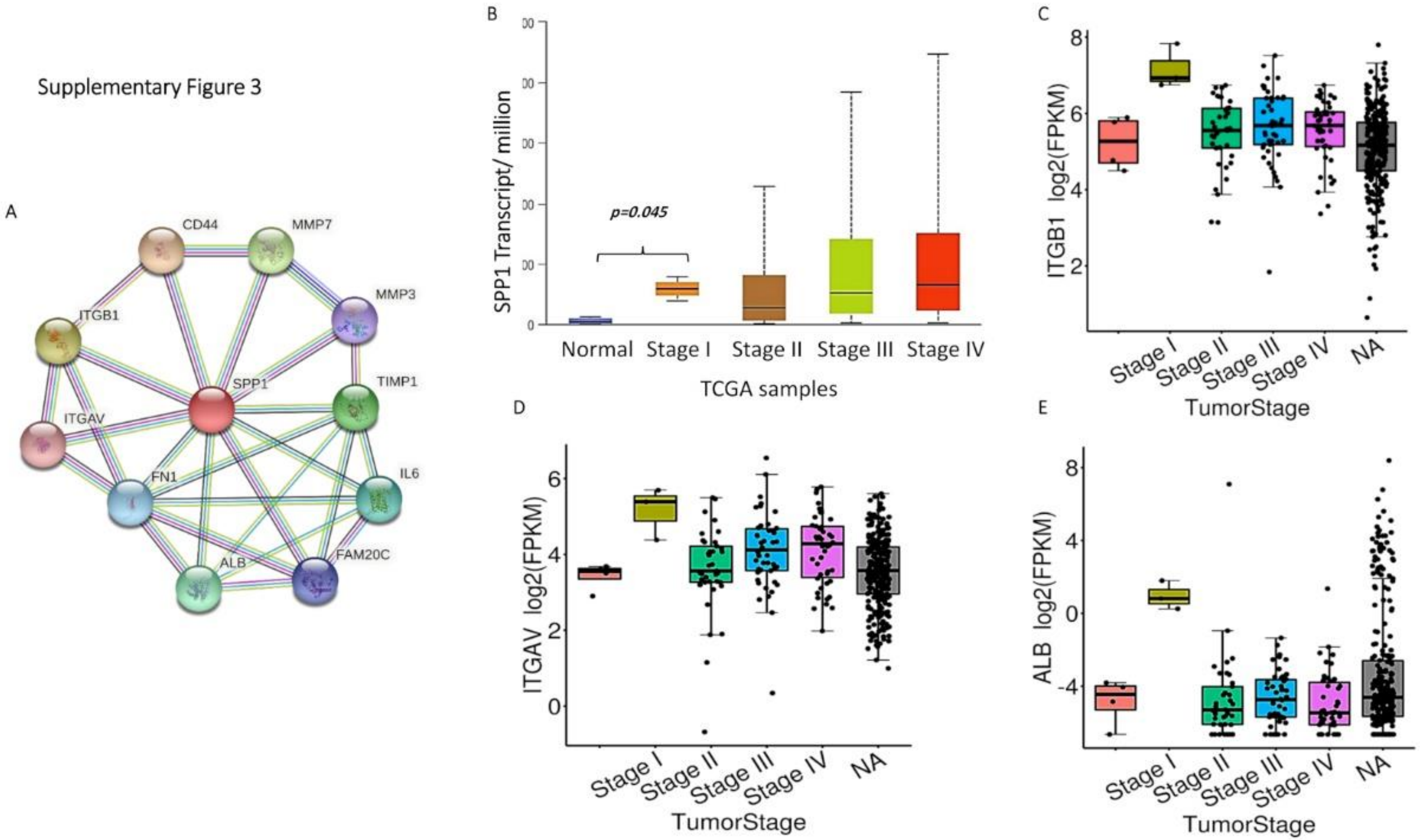

Supplementary figure S3: Functional interaction of SPP1 in bladder cancer. (A) Predicted protein-protein interaction network for SPP1 as illustrated in the STRING database. Box plots determining the correlation between the expressions of SPP1-interacting proteins: SPP1 (B), integrin beta ITGB1 (C), integrin alpha ITGAV (D) and albumin ALB (E) based on different stages of bladder cancer development. Data was explored in TCGA-BLCA project using UALCAN database.
